# Is the anterior cervical dynamic plate fixation better than the anterior static plate fixation: a retrospective review with over 5 years follow-up

**DOI:** 10.1186/s12891-023-06156-9

**Published:** 2023-01-18

**Authors:** Chao Li, Qing He, Yue Zhu, Zuqiang Wang

**Affiliations:** 1 Department of Orthopedics, The Sixth Medical Center, General Hospital of Chinese PLA, Beijing, 100048 China; 2 Department of Wound Repair and Rehabilitation Medicine, Center of Bone Metabolism and Repair, State Key Laboratory of Trauma, Burns and Combined Injury, Trauma Center, Research Institute of Surgery, Daping Hospital, Army Medical University, Chongqing, China; 3 Navy Clinical College, Anhui Medical University, Hefei, 230032 Anhui Province China

**Keywords:** Cervical, Dynamic plating, Static plating, ACDF

## Abstract

**Background:**

To compare the clinical and radiologic outcomes after anterior cervical dynamic or static plate fixation for short segment cervical degenerative disc diseases (DDD) for more than 5 years.

**Methods:**

Sixty-four patients who underwent anterior cervical one level discectomy or corpectomy with an anterior cervical plate system were followed for an average of 6.8 years for clinical and radiographic outcomes. Among the sixty-four patients, thirty-eight patients were fixed with a static plate (ORION and CSLP plate system) and the other twenty-six patients were fixed with a dynamic plate (ABC plate). Radiographic data were collected included the global sagittal alignment of the cervical spine (C2–C7), the local height and angle of the operated level pre-operatively, postoperatively and at last follow-up. A clinical assessment was performed at pre-operatively, three months postoperatively and final follow-up using the Japanese Orthopedic Association (JOA) /Visual Analogue Score(VAS)/ Neck Disablility Index(NDI) scoring system.

**Results:**

The mean follow-up time was 6.8 years. At final review, there were two cases of suspicious pseudarthrosis which were from ABC plate fixation group while the other cases all gained solid fusion. The height of fusion segment gained significantly improvement for both dynamic and static plate group post-operation, and all groups demonstrated a significant loss in height postoperatively. Generally, for the one level ACDF group, the height decrease was 0.5 mm for static plate and 1.6 mm for dynamic group which was significantly different(*p* < 0.05). And for one level ACCF group, this type of difference was not seen in which decreasing was 1.7 mm for static group and 1.8 mm for dynamic group. Segmental lordosis of the fusion segments was increased significantly both post-operation and final follow-up than before-operation for both one and two segments fusion. Global cervical lordosis from C2–C7 was increased in the early postoperative period in all groups, and at final follow-up the total lordosis was still getting better compared with early postoperative period, but this increase was not statistically significant. Clinical assessment of JOA/NDI showed that there was significantly improvement 3-month post-operation compared with pre-operation, and the score could get a slight further improvement at the final follow-up.

**Conclusion:**

Our study demonstrated a statistically similar fusion rate between dynamic and static cervical plate fixation. However, the height gained with static plate fixation for single segment disease was maintained better than with dynamic plate fixation and there was no difference between JOA outcome scores between groups. Despite the reported improved biomechanics of dynamic plate fixation, further research needs to be done to show the clinical advantage of dynamic plate fixation.

## Background

Anterior cervical discectomy and fusion (ACDF) or anterior cervical corpectomy and fusion (ACCF) with plate fixation is now the standard of care for patients with cervical degenerative disc disease. Besides gaining stability, the use of plates has been demonstrated to provide improved fusion and deformity correction [1]. Since the first anterior plating attempted for cervical trauma in 1964, there has been an evolution in plating systems for the anterior cervical spine [2]. Constrained or static plates, such as CSLP (Synthes, West Chester, Pennsylvania, USA) and Orion (Medtronic Sofamor Danek, Minneapolis, Minnesota, USA) plates, have a mechanism for locking for more safe, but these plates are thought too rigid and may prevent the interbody graft from receiving the necessary loads for fusion [3]. Dynamic plate permits for limited vertical motion and settling, which is a potential solution for improving fusion rates. A long-term study comparing these two plate fixation systems has not been previously reported. There are both static and dynamic fixation plate systems but with no comparative study to date. Compared to a static plate, the dynamic plate has theoretically biomechanical advantage with greater stress distribution favoring further fusion. However, it is not clear whether this biomechanical difference will affect the clinical and radiological outcomes.

This study compared the long-term radiologic and clinical outcomes between patients following one-level ACDF or ACCF with static or dynamic plate fixation. Patients were assessed for: 1) clinical improvement, 2) fusion rates, 3) global and focal sagittal alignment, and 4) graft subsidence.

## Methods

### Patients

**Fig. 1:**
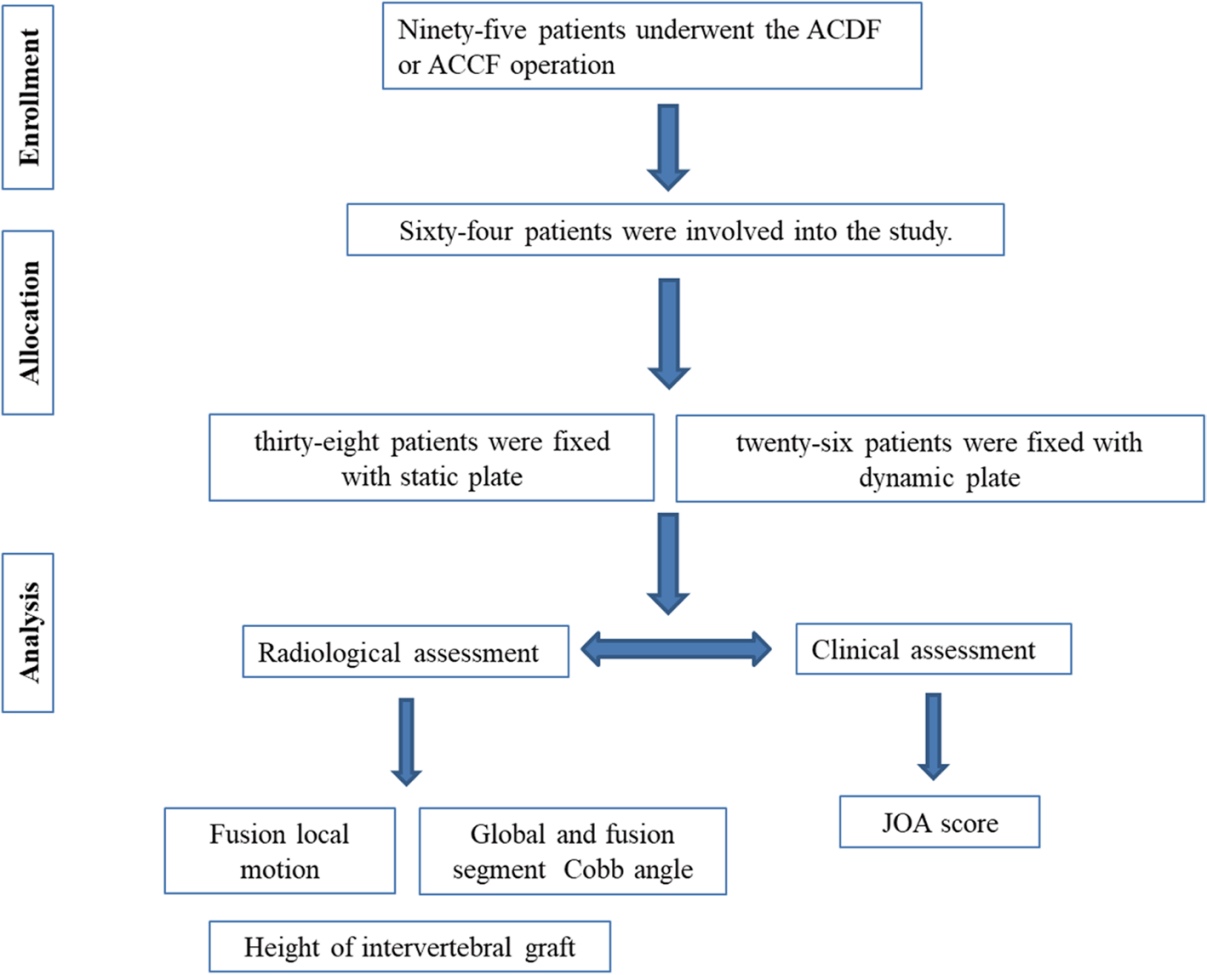
CONSORT flow diagram of patients included in this investigation

A retrospective review was performed of patients at our institution between January 1, 2015 and December 31, 2016 who underwent a one-level ACDF or ACCF with either static or dynamic plate fixation. The static plates used were Orion (Medtronic Sofamor Danek, Minneapolis, Minnesota, USA) and CSLP (Synthes, West Chester, Pennsylvania, USA), and the dynamic plate used was an ABC plate (Aesculap, Center Valley, Pennsylvania,USA). Finally, Sixty four of 115 patients returned for follow-up, 36 males and 28 females, with an average of age of 58.4 years (range, 36–72 years). The levels fused for ACDF were C4-5(30 patients), C5-6(10patients) and C3-4(2 patients). The levels fused for ACCF were C5 (15 patients), C4 (5 patients) and C6 (2 patients). The pathology included cervical spondylotic myelopathy (48 patients, 75%), cervical disc herniation (8 patients, 12%), OPLL (6 patients,10%) and cervical trauma (2 patients, 3%). Figure 1 shows a chart of the trial design. The Medical Board Ethics Committees of the Six Medical Center of PLA General Hospital (Beijing, China) approved the study (IRB number HZKY-PJ-2016–18).

### Surgical technique

**Fig. 2:**
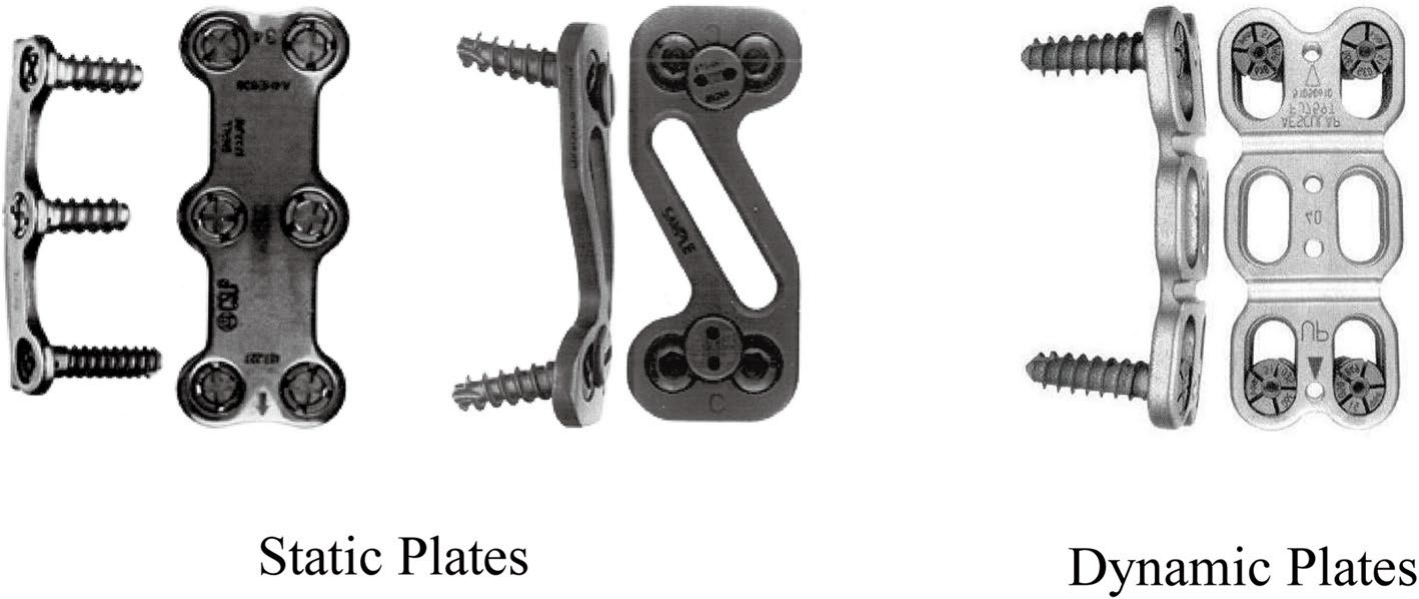
Introduction of the static or dynamic plate fixation. Static plate: Orion (Left) and CSLP plate (Right), and the dynamic plate

**Fig. 3:**
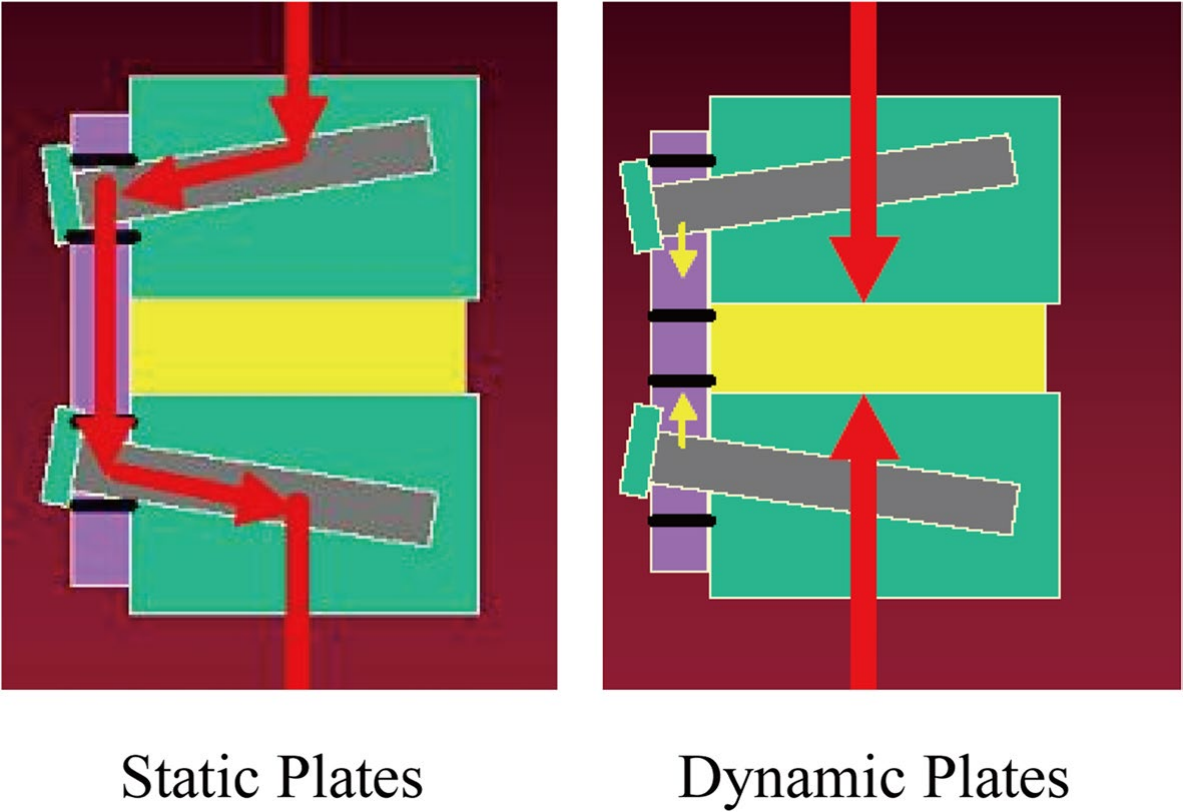
Stress conduction pattern of anterior cervical plate. For the static plates, most of the stress is transmitted through the plate (left), and for the dynamic plates, most of the stress is transmitted through the bone graft (right)

A standard right side Smith Robinson approach was used for all patients. A discectomy was completed in all cases with Caspar pin distracter by removing both the disc material and the posterior longitudinal ligament. For the cases with huge osteophyte compression at the posterior edge of the vertebral body, single level decompression and high risk of spinal cord injury, as well as the cases with ossification of the posterior longitudinal ligament of the cervical spine and fracture of the cervical vertebral body, the ACCF method is generally selected. For cases which adequate decompression of the posterior border of the vertebral body can be achieved by simple discectomy, ACDF could be chosen. In cases of a corpectomy, curet and/or a high-speed burr was used. The posterior longitudinal ligament was removed in all cases. Tricortical anterior iliac crest for autograft harvested with a low-speed oscillating saw was used in all cases and inserted into the defect space with the cortical surface facing anteriorly. The appropriate length plate was chosen and screw fixation was placed just beneath the respective endplate. In this area not only screws can gained the mostly strength fixation but also can avoid the invading to the adjacent disc for the dynamic plate after screws sliding (Figs. 2 and 3). Postoperative immobilization consisted of a soft collar for 4 to 6 weeks. Radiographs were regularly taken at 3, 6 and 12 months after surgery to assess for fusion and for the presence of any implant or graft failure, and the last radiographs were taken for the final follow-up.

### Outcomes assessment

**Fig. 4:**
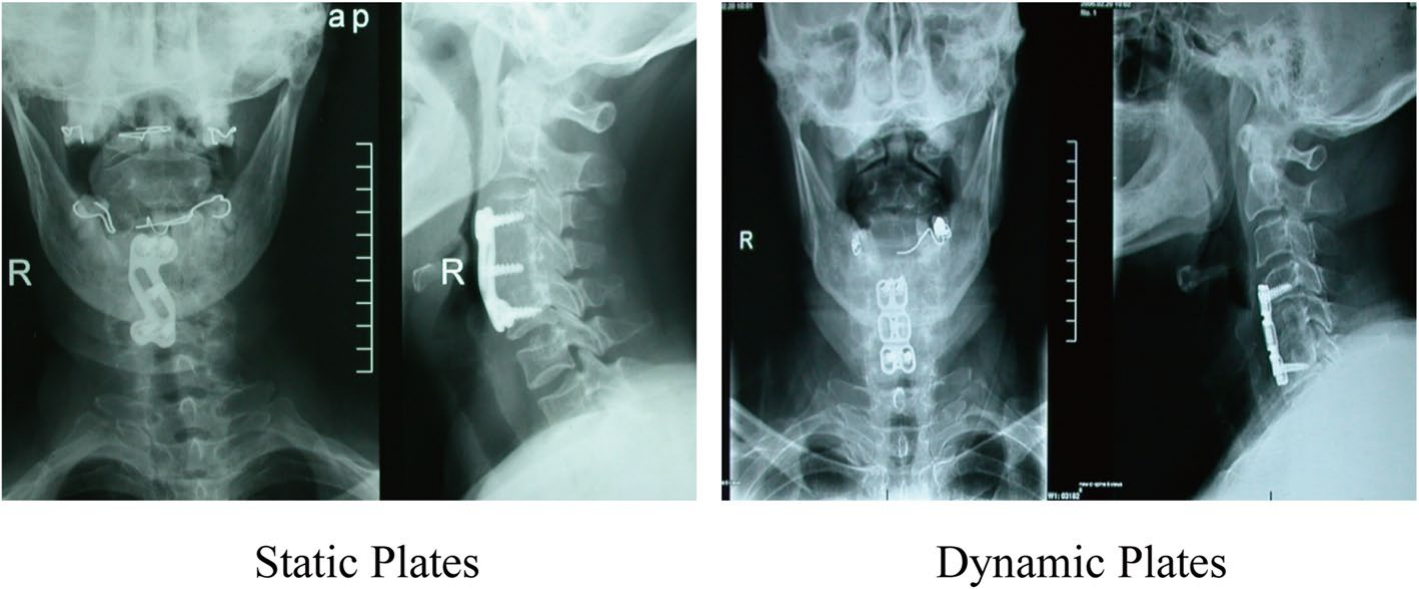
X-ray view of anterior cervical corpectomy and fusion post operation 2 years

The cervical sagittal parameters of C2-7SVA (sagittal vertical axis), C2-7 and the operated levels Cobb’s angle were measured before and after surgery and follow-up times (Fig. 4). To evaluate the cervical sagittal, the height of the intervertebral graft was measured as the distance between the mid-point of the upper and lower end plate of the cranial and caudal vertebral body respectively. For suspected fusion cases, two-dimensional computed tomography was used.

Radiographic fusion was defined as: absence of motion between the spinous processes (less than 2 mm) and/or intervertebral body (less than 2° for Cobb’s angle) on flexion–extension radiographs; absence of lucent lines at the interface between the graft and the adjacent vertebral endplates; and the presence of bridging bony trabeculae at the graft-endplate junction [4].

Clinical assessment was performed before surgery, three months after operation and at final follow-up on using the Japanese Orthopaedic Association (JOA) scoring system including the motor and sensory status of the upper and lower limbs and bladder function [5].

### Statistical analysis

Data were analyzed by GraphPad Prism v.6.01 software (GraphPad Inc., La Jolla, CA, USA). All patients were divided into ACDF and ACCF group, and the patients in ether group were fixed with dynamic or static plate. The analysis of variance test was used to compare changes in height of intervertebral graft and segmental and global cervical lordosis before surgery, in the early postoperative period and at final review for each group. The same method was used to compare spinal cord functional recovery with the JOA/ VAS/NDI scores. Statistical significance was set as a *P* value less than 0.05.

## Results

The mean follow-up time was 6.8 years. Totally there was 115 patients accepted one level ACDF or ACCF, and 76 patients’ response to the follow-up study. Among the 76 patients, there were 64 patients with adequate film records including pre-operation, post-operation and final follow-up X-rays. Of the 64 patient who had adequate films available for the analysis thirty-eight patients were fixed with static plate, including Orion (24 cases) and CSLP (14 cases), and the other twenty-six patients were fixed with a dynamic plate of ABC. There were 42 patients who underwent a one-level ACDF including 26 with a static plate and 16 with a dynamic plate. And the other 22 patients underwent a one-level ACCF fixed with a static plate (12 patients) or dynamic plate (10 patients). All grafts were harvested from autologous iliac crest bone. The follow-up interval included films obtained at 3, 6,12 months and the last follow-up. Except for the post-op at 3 month all films included anterior–posterior, lateral and flexion and extension view films (Tables 1 and 2).

**Table 1:** The patient’s demographic pre-operative data

	Gender		Type of disease				Total
	Male	Female	CSM	Cervical disc herniation	OPLL	Trauma	
Static Plate	21	17	28	4	4	2	38
Dynamic Plate	15	11	20	4	2		26
Total	36	28	48	8	6	2	64

**Table 2:** Distribution of cases

Operation Type	Level	Static Plate(N)	Dynamic Plate(N)	Total
ACDF	C3-4	2		42
	C4-5	16	14	
	C5-6	6	4	
ACCF	C4	4	1	22
	C5	9	6	
	C6	1	1	
Total		38	26	64

There was no incidence of graft extrusion or migration. There were two radiological non-unions confirmed by CT scan half a year post-operation which were from dynamic and static plate group of ACCF, however, neither patient reported symptoms, and the fusion was confirmed one year later which was delayed fusion. There were three cases of a hematoma at the graft site for the iliac crest, and treated successfully with physical therapy. Five patients reported prolonged (greater than 24 months) iliac crest graft site pain.

There were no implant complications of plate breakage, screw loosening and withdrawal et.al. All cases demonstrated a significant restoration of height after surgery (Table 3). All groups demonstrated a significant loss in height postoperatively. Generally, for the one level ACDF group, the height decrease was 0.5 mm for a static plate and 1.6 mm for dynamic group which was significantly different(*p* < 0.05). And for one level ACCF group, this type of difference was not seen in which decreasing was 1.7 mm for static group and 1.8 mm for dynamic group. Although there was height decreasing, the height of any fusion segment was still higher than pre-operation significantly.

**Table 3:** Radiological data

	Height of affected segment (mm)			Intervertibral angle of affected segment (degrees)			Global sagital cervical Cobb angle in neutral position (degrees)			C2-7 SVA (mm)			Fusion rate (half a year post-op)
	Before operation	After operation	At final follow-up	Before operation	After operation	At final follow-up	Before operation	After operation	At final follow-up	Before operation	After operation	At final follow-up	
ACDF													
Static(24)	35.5 ± 6.7	38.4 ± 6.7^*^	37.9 ± 4.4^△^	1.2 ± 5.6	7.6 ± 3.6^*^	7.0 ± 3.4^*^	5.8 ± 2.9	12.5 ± 4.8^*^	14.1 ± 5.1^*^	15.5 ± 6.4	18.2 ± 8.7*	21.5 ± 11.2*^△^	100%
Dynamic(18)	36.8 ± 0.7	39.1 ± 0.8^*^	37.0 ± 1.3^△^	2.5 ± 7.7	10.4 ± 4.6^*^	8.5 ± 5.1^*^	6.0 ± 2.8	13.0 ± 5.3^*^	13.6 ± 4.5^*^	17.8 ± 8.1	22.5 ± 9.6*	24.3 ± 11.0*	100%
ACCF													
Static (14)	53.8 ± 5.8	57.9 ± 8.1^*^	56.2 ± 6.7	6.4 ± 2.3	8.3 ± 2.1^*^	8.3 ± 2.3^*^	5.1 ± 1.9	12.9 ± 4.4^*^	14.6 ± 5.3^*^	13.2 ± 5.9	17.5 ± 8.4*	19.5 ± 12.6*	92.9%
Dynamic(8)	53.8 ± 5.5	57.9 ± 6.0^*^	56.1 ± 5.5	6.7 ± 6.6	9.4 ± 4.6^*^	8.9 ± 1.6^*^	4.8 ± 1.9	13.5 ± 5.0^*^	13.7 ± 5.1^*^	11.4 ± 5.8	16.5 ± 7.4*	20.5 ± 10.3*^△^	87.5%

The descriptive data represents mean ± standard deviation (mm or degree)

^*^indicates a significant (*p* < 0.05) difference between pre-operative and post-operative values

^△^indicates a significant (*p* < 0.05) difference between groups post-operatively

Segmental lordosis of the fusion segments was increased significantly post-operation than pre-operation for both one and two segments fusion (Table 3). Although some of the lordosis restored was lost by final review, this was still significantly better than before operation. Global cervical lordosis from C2–C7 was increased in the early postoperative period, and at final follow-up the total lordosis was still getting better compared with early postoperative period, but this increase was not statistically significant. The same trend can be observed in C2-7 SVA measurement.

Clinical assessment of Japanese Orthopedic Association (JOA) scoring system showed that there was significantly improvement after operation 3 m compared with before operation, and the JOA score get a little better at the final follow-up than post-operation, but there wasn’t significant difference (Table 4). Overall, clinical symptoms of myelopathy and radiculopathy significantly improved after surgery. VAS score and NDI score reflecting neck symptoms showed that the neck function immediately after surgery was still limited compared with that before surgery, and the neck function status was significantly improved at the 3-month follow-up (Tables 5 and 6).

**Table 4:** Clinical assessment of JOA score

	Preoperatively	Postoperatively	Follow-Up
Static Plate group	10.45 ± 3.32	12.63 ± 1.70^*^	14.37 ± 2.06^**^
Dynamic Plate group	11.50 ± 3.17	13.12 ± 2.12*	15.10 ± 1.91**

*JOA* Japanese Orthopaedic Association

^*^Indicates a significant (*p* < 0.05) difference between pre-operative and post-operative values

^**^Indicates a significant (*p* < 0.05) difference between follow-up and post-operative values

**Table 5:** Clinical assessment of VAS score

	Preoperatively	Postoperatively	Follow-Up
Static Plate group	4.6 ± 1.9	5.5 ± 1.70	1.6 ± 0.6*
Dynamic Plate group	5.1 ± 2.0	5.3 ± 2.2	1.5 ± 0.5*

*VAS* Visual Analogue Score

^*^Indicates a significant (*p* < 0.05) difference between pre-operative and post-operative values

^**^Indicates a significant (*p* < 0.05) difference between follow-up and post-operative values

**Table 6:** Clinical assessment of NDI score

	Preoperatively	Postoperatively	Follow-Up
Static Plate group	(55 ± 18)%	(31 ± 17) %*	(15 ± 5)% **
Dynamic Plate group	(61 ± 22)%	(27 ± 14)% *	(13 ± 6)% **

*NDI* Neck Disablility Index

^*^Indicates a significant (*p* < 0.05) difference between pre-operative and post-operative values

^**^Indicates a significant (*p* < 0.05) difference between follow-up and post-operative values

## Discussion

During the past decades anterior cervical fusion with plate has gained wider acceptance and undergone. Anterior cervical spinal decompression and fusion techniques were initially popularized in the 1950s by Bailey and Badgley, Smith and Robinson, and Cloward [6, 7]. The use of the plate decreases the micro motion across the fusion construct and provides stability necessary for successful fusion to take place. It has been accepted that anterior cervical plating can improve fusion rates after the ACDF. Wang [8] had reported a 0% nonunion for two-level ACDF with the use of anterior cervical plating.

The first-generation anterior cervical plating system was developed by Orozco and Llovetand Caspar [2] et al. which was now also termed non-constrained plates because the screw was not locked to the plate, so for more screw grasp strength the bicortical screw was needed which was directly risk to spinal cord. The second generation was locked-screw plate which has a mechanism to secure the screws within the plate. Although the mechanism of these plates can prevent screw back out, these plates have been criticized for being too rigid and the interbody graft was shielded from receiving axial loads stress for fusion. The third generation of plates, called dynamic plates, had mechanisms to allow for loads to be carried through the interspace and graft, including semi-constrained rotational plates and semi-constrained translational plates. The theoretical advantage of dynamic plates is greater graft loading with less stress shielding, which should increase fusion rates [9].

Brodke et al. [10] compared static, rotationally dynamic, and translationally dynamic plates in a single-level corpectomy cadaveric model in a biomechanical study. They showed that when a 10% subsidence of the interbody spacer occurred, the load-sharing acrossing the interspace decreased significantly when a static plate was used, but was maintained largely with both the rotationally dynamic and translationally dynamic plates. And the stability biomechanical test by Dvorak et al. [11] showed no significant difference in the range of motion with the two plates, except for extension where the dynamic plate allowed less motion.

Although dynamic plate has the biomechanical advantage, clinical study didn’t give the same consequence. Multiple studies have reported the results of different types of plating systems, however, there is not sufficient evidence to support one type of plate over another [12, 13]. In our study, the two cases of un-fusion were belonging to the dynamic group. In contrary, all cases in static group gained well fusion. The judge of pseudarthrosis still is difficult. Usually, the pseudarthrosis or fusion was judged by overall presence or absence of motion between spinous processes, presence or absence of radiolucency at the graft-endplate junction, and evidence of bridging between the endplate and the graft. In our two cases the only found was the radiolucency at the graft-endplate junction without the motion between spinous processes. A number of studies have shown that the presence of a pseudarthrosis correlates with poorer clinical outcomes [3]. But the two cases in our study didn’t show special symptom on the neck compared with the fusion ones.

In our study, the fusion rate of the static plate was slightly higher than the dynamic plate, but this finding was not statistically significant. Thus, it may be stated that the fusion rate of one- or two-level segment is so high that we can’t detect the difference between two types of plate, especially with the autograft. In a similar study, Goldberg et al. [14] compared fusion rate with the static and dynamic plate with allograft in two-level anterior cervical discectomy using a retrospective review for averaged follow-up time period of 10 months. The study showed that the rate of fusion with a dynamic plate was similar to that of the static plate despite the use of allograft bone with the dynamic plate. In their 10 to 13 month interval, the fusion rate had increased to 84.7% for the static plate/autograft group and 90% for the dynamic plate/allograft group.

Subsidence is popular phenomenon for two types of plate. The biomechanical character of compression of dynamic plate can be seen at one level segment fusion. The subsidence for static group was 0.5 mm, and the dynamic group was 1.6 mm which was significantly different. But in two level fusion group, we found there wasn’t this type of difference which subsidence were 1.7 mm and 1.8 mm for static and dynamic plate separately. Maybe the length of subsidence was depended on the sum of fusion graft-bone interface. For corpectomy, the number of graft-bone interface was same with discectomy. When fusion with autograft, the subsidence was limited and didn’t develop a segmental kyphosis over the operative levels.

It must be mentioned that more technical was needed for dynamic plate. Length suitable plate must be chosen to ensure there will be no invadation to the adjacent disc after screws sliding in the slot. It was reported that in some cases the slotting was so obviously that when fusion the edge of plate had connected with the adjacent disc which was potential to induce the degeneration of that disc [15]. In our study we didn’t find the case which slotted so much case because of limitation of subsidence.

So for short segment cervical fusion both static and dynamic plate can gained good fusion, although the dynamic plate has the advantage of biomechanics. A number of studies have reported decreased fusion rates for the multilevel discectomy and fusion. Whether the dynamic plate can do better in long segment fusion need to study further.

## Conclusion

Our study showed that a statistically similar fusion rate between dynamic and static cervical plate fixation. We also found that the height gained with static plate fixation for single segment disease was maintained better than with dynamic plate fixation and there was no difference between JOA/VAS/NDI outcome scores between groups. Despite the reported improved biomechanics of dynamic plate fixation, further research needs to be done to show the clinical advantage of dynamic plate fixation.

## Data Availability

The datasets generated during and analyzed during the current study are not publicly available due to the protection of patient privacy but are available from the corresponding author on reasonable request.
